# The Public Service Motivation’s Impact on Turnover Intention in Korean Public Organizations: Do Perceived Organizational Politics Matter?

**DOI:** 10.3390/bs15040474

**Published:** 2025-04-06

**Authors:** Jae-Young Lim, Kuk-Kyoung Moon

**Affiliations:** 1 Department of Public Administration and Social Welfare, Chosun University, Gwangju 61452, Republic of Korea; jaeyounglim@chosun.ac.kr; 2 Department of Public Administration, Inha University, Incheon 22212, Republic of Korea

**Keywords:** public service motivation, perceived organizational politics, turnover intention

## Abstract

Increasing turnover intention among public employees in Korean public sector organizations endangers both organizational sustainability and public service quality. Although prior research highlights job stress, compensation systems, and organizational culture as key drivers of turnover, scholars in limited empirical studies directly examine the role of public service motivation. In this study, we address this gap by investigating whether public service motivation reduces turnover intention and how perceived organizational politics may moderate this relationship. Using survey data from the 2023 Korean Public Employee Viewpoints Survey—conducted by the Korea Institute of Public Administration and including responses from central and local government employees—we employ stereotype logistic regression for analysis. Results show that public service motivation significantly lowers turnover intention, but its positive effect diminishes when employees perceive high levels of organizational politics. When employees believe that power and resources are distributed based on political interests rather than merit, they experience diminished reciprocity toward their organization. As a result, their intention to leave the organization increases. These findings underscore the need to sustain and enhance public service motivation while mitigating perceived organizational politics. Enhancing fairness, transparency, and trust—while reducing political interference—can preserve public service motivation’s positive impact and reduce turnover intention within public sector organizations.

## 1. Introduction

Public employee turnover has emerged as a critical challenge for government organizations worldwide, including South Korea, reflecting a broader global trend that threatens the stability and quality of public service delivery (Hur & Abner, 2024). Traditionally, public service was regarded as a stable career with relatively low turnover rates (Chung et al., 2022). However, an increasing number of public employees have recently considered leaving their positions, signaling deeper structural and organizational challenges rather than mere individual dissatisfaction (Cohen et al., 2016; Jung, 2010). These challenges include declining public-sector values, administrative instability, and growing employee dissatisfaction (Jeon, 2024; Yun et al., 2019). Moreover, increasing skepticism about the core values of public service—such as fairness in decision-making and commitment to serving the public interest—as well as doubts regarding the broader mission of government work require urgent attention (Kyeong & Kim, 2022). Employees who perceive that their organizations fail to uphold these values may experience diminished motivation and professional disengagement, potentially further accelerating turnover trends (Perry & Wise, 1990). In addition to these value-based concerns, many public employees cite low compensation, excessive workloads, rigid hierarchical structures, slow promotions due to seniority-based systems, and fatigue from frequent policy changes as key drivers of turnover (Kaufmann et al., 2019; Rainey, 2014).

Beyond these structural challenges, the perception of fairness and trust within organizations plays a significant role in employee retention (Colquitt et al., 2013). Employees who perceive decision-making processes as unfair, career advancement opportunities as limited, or political favoritism as prevalent may experience reduced job satisfaction, weakened organizational commitment, and ultimately, higher turnover intentions (Aryee et al., 2004; Vigoda-Gadot, 2007). Given the significant financial and operational costs associated with high turnover—including the loss of institutional knowledge, declining service quality, increased administrative burdens, and rising recruitment and training expenses—addressing turnover intentions has become a strategic priority for governments worldwide, not just in South Korea (Pitts et al., 2011; Sun & Wang, 2017).

Public service motivation, defined as an individual’s intrinsic motivation to contribute to the public good beyond personal financial gain (Perry & Wise, 1990), has been widely recognized as a key factor in public employee retention (Bright, 2008; Campbell & Im, 2016). Employees with high public service motivation generally experience greater job satisfaction, a stronger sense of purpose, and a higher level of organizational commitment, thereby reducing turnover intentions (Awan et al., 2020). Many scholars suggest that individuals who strongly identify with public service values are more willing to endure challenges such as bureaucratic inefficiencies and lower salaries if they perceive their work as meaningful (Homberg et al., 2015). However, recent research challenges the assumption that public service motivation alone is sufficient to ensure employee retention. Structural conditions such as low compensation, high workloads, limited career progression, and lack of recognition can erode motivation over time, leading to frustration and eventual disengagement (Maslach & Leiter, 2008). Empirical studies have shown that without supportive workplace conditions, even highly motivated public employees may experience burnout, cynicism, and increased turnover intentions (Ballart & Ripoll, 2024; Camilleri, 2007; Fernandes et al., 2022).

Even when structural conditions are relatively favorable, employees’ perceptions of organizational politics can further diminish the positive effects of public service motivation on retention (Park & Lee, 2020). Perceived organizational politics refers to employees’ beliefs that favoritism, self-interest, or informal networks rather than meritocracy and fairness influence decision-making, promotions, and resource allocation within their organization (Ferris & Kacmar, 1992). Employees who perceive a highly political work environment may feel that their contributions are undervalued, their career prospects uncertain, and their efforts to serve the public good obstructed by bureaucratic or politically motivated decisions (Meisler & Vigoda-Gadot, 2014). This undermines the psychological contract between employees and their organization, weakening the expected reciprocal relationship between motivation and organizational support (Morrison & Robinson, 1997). Thus, in this study, we hypothesize that perceived organizational politics does not merely coexist with structural challenges but actively moderates the relationship between public service motivation and turnover intention. This moderation may potentially neutralize the positive effects of public service motivation in highly political environments.

This study applies social exchange theory, which posits that employees who perceive fair recognition and rewards will reciprocate with higher commitment and lower turnover intention (Blau, 1964). However, in organizations where political factors override performance-based rewards and career advancement, employees may feel a lack of control over their professional growth, leading even highly motivated individuals to reconsider their long-term commitment (Colquitt et al., 2013; Kacmar et al., 2011). Studies have shown that public employees working in organizations with high levels of perceived political interference report lower job satisfaction and higher turnover intentions, particularly in bureaucratic environments where career advancement often depends on subjective assessments rather than objective performance metrics (Chang et al., 2009; Vigoda-Gadot, 2000). Thus, even among employees with strong public service motivation, perceptions of political favoritism may erode their sense of fairness, weaken organizational commitment, and ultimately increase turnover intention. By explicitly examining the moderating role of perceived organizational politics, this study provides a more nuanced understanding of how the interplay between motivation and organizational conditions influences turnover intention.

Given these challenges, this study addresses the following research questions: (1) Does public service motivation reduce turnover intention among public employees? and (2) To what extent do perceptions of organizational politics moderate this relationship? Using data from the 2023 Korean Public Employee Viewpoints Survey, we find that public service motivation significantly reduces turnover intentions. However, its positive effects are substantially weakened when employees perceive high levels of organizational politics. These findings underscore the importance of fostering organizational environments characterized by transparency, fairness, and meritocratic practices to leverage public service motivation for employee retention effectively.

In this study, we contribute to the literature by empirically examining how organizational politics moderates the relationship between public service motivation and turnover intention, addressing a gap in previous research that primarily focused on direct effects of public service motivation. Unlike previous scholars, who primarily examined the direct effects of public service motivation on turnover intention, we advance the field by empirically testing the moderating role of organizational politics using large-scale public sector survey data. This research also highlights that public service motivation alone is insufficient to ensure employee retention, underscoring the necessity of fair and transparent organizational conditions. Finally, although this study focuses on South Korea, its findings are relevant to other national contexts where bureaucratic inefficiencies, political favoritism, and employee retention challenges similarly impact public-sector organizations. The study’s insights can inform strategies for improving transparency, fairness, and motivation-driven retention in governments with comparable administrative structures.

To address these research questions, we adopt a structured approach. First, we review existing research on public service motivation, turnover intention, and organizational politics to establish a theoretical foundation and develop hypotheses. Second, we conduct an empirical analysis using data from the 2023 Korean Public Employee Viewpoints Survey, applying stereotype logistic regression, a statistical method suited for examining categorical dependent variables, to test the proposed hypotheses. Finally, we discuss its practical and theoretical implications, highlighting how the findings contribute to both academic discourse and public sector management practices. Specifically, we offer strategies for strengthening public service motivation while mitigating the negative effects of organizational politics, ultimately providing insights for policymakers and administrators seeking to improve employee retention and organizational effectiveness.

## 2. Theoretical Background and Hypotheses

### 2.1. The Impact of Public Service Motivation on Turnover Intention

Public service motivation is a key concept that explains the intrinsic motivation of those working in the public sector, and scholars across various academic fields have examined it extensively (Christensen et al., 2017; Ritz et al., 2016). Unlike traditional motivational theories that focus primarily on personal gains, public service motivation emphasizes the importance of public interest and communal well-being. Perry and Wise (1990) systematically developed public service motivation as a unique motivational factor for public officials, and subsequent research has built on this framework, offering in-depth analyses of how public service motivation helps create social value and achieve the public good. At the organizational level, numerous studies have empirically demonstrated public service motivation’s positive effects on employees’ attitudes, job satisfaction, and performance (Harari et al., 2017; Tang et al., 2024). More recently, scholars have examined how individual and organizational characteristics interact to shape and strengthen public service motivation, solidifying its status as a crucial framework for understanding motivation and organizational behavior in the public sector (Crucke et al., 2022; Hameduddin & Engbers, 2022).

Public service motivation, defined as an individual’s intrinsic drive to prioritize societal interests over personal gains (Kim, 2009; Perry & Wise, 1990), forms the basis for our first hypothesis regarding its direct effect on turnover intention. This intrinsic motivation, rooted in a sense of duty to serve the public good, is expected to reduce employees’ intention to leave their organizations. A core characteristic of public service motivation is the willingness to make personal sacrifices for the sake of societal benefit, whether through long working hours, lower salaries, or the pursuit of policy goals that prioritize public welfare over private interests (Brewer et al., 2000; Perry, 1996). Employees with high public service motivation tend to experience greater job satisfaction and organizational commitment, which in turn contribute to lower turnover intentions and enhanced public service quality (Kim, 2012).

Turnover intention, defined as an employee’s consideration of leaving their organization, is a key predictor of actual turnover and has significant implications for public-sector stability and service quality (Cohen et al., 2016). Research identifies job satisfaction, burnout, perceived fairness, and leadership style as primary antecedents, with dissatisfaction, excessive workloads, and lack of career growth increasing turnover intention (Chordiya, 2022; Hur & Abner, 2024). Organizational factors such as rigid hierarchies, bureaucratic inefficiencies, and limited merit-based advancement further contribute to turnover (Jung, 2010). Additionally, external factors, including labor market conditions and generational differences, influence employees’ willingness to stay (Lyons et al., 2015). High turnover leads to the loss of institutional knowledge, increased recruitment costs, and weakened public trust in government institutions (Meier & Hicklin, 2008). Given these consequences, understanding how public service motivation interacts with these organizational dynamics to shape turnover intention is essential for improving employee retention in public organizations.

Given that public service motivation has been widely linked to reduced turnover intention, previous scholars have examined its three primary dimensions—rational, normative, and affective motivation (Perry & Wise, 1990). Each dimension contributes differently to employee retention: the rational dimension through policy engagement, the normative dimension through ethical commitment, and the affective dimension through emotional connection to public service. First, the rational dimension of public service motivation reflects a desire to engage actively in policymaking to create social change (Vandenabeele, 2007). Employees with high rational motivation view their work as an opportunity to enhance policy efficiency, support marginalized groups, and implement meaningful reforms (Brewer et al., 2000). Because they perceive their roles as integral to public service delivery, they are more likely to remain committed to their organizations as long as structural barriers do not impede their efforts (Vandenabeele, 2007). Second, the normative dimension of public service motivation is rooted in ethical and moral convictions that emphasize public service as a duty. Employees with high normative motivation feel a strong sense of responsibility toward their organization and society (Houston, 2000). When they believe their work aligns with ethical principles—such as fairness, social justice, and integrity—they are more likely to remain committed to their organization (Vandenabeele, 2007). However, when they perceive misalignment between organizational actions and public values, their motivation may weaken, increasing turnover risk. Finally, the affective dimension of public service motivation relates to emotional commitment to helping others. Employees with strong affective motivation often feel empathy toward marginalized populations and take pride in work that improves social welfare (Kim, 2012). This emotional connection enhances their professional identity and job satisfaction, further reducing turnover intentions (Ritz et al., 2016). When employees feel their work has a meaningful societal impact, they are more likely to remain in their positions despite bureaucratic challenges.

Numerous previous studies have consistently demonstrated that public service motivation has a direct negative relationship with turnover intentions within the public sector. For instance, Shim et al. (2017), using a sample of 4974 Korean street-level bureaucrats, found that higher public service motivation levels correspond with a lower likelihood of leaving their organization, as these individuals’ intrinsic motivation to serve the public closely aligns with their roles and responsibilities. Similarly, Campbell and Im (2016) found that public service motivation nurtures a deep commitment to public sector values, directly lowering employees’ intentions to quit. Building on these insights, Wang et al. (2020) confirmed the direct negative link between public service motivation and turnover intention in a study of 587 public employees in Yunnan Province, China. Their findings highlighted how employees with elevated public service motivation demonstrate a reduced inclination to leave due to their strong intrinsic alignment with public service values. Collectively, these findings emphasize public service motivation’s indispensable role in directly curtailing turnover intentions by reinforcing employees’ sense of purpose and commitment to public sector roles. Based on this, we propose the following hypothesis:

**Hypothesis 1:**  *Public service motivation has a negative relationship with turnover intention.*

### 2.2. Moderating Impact of Perceived Organizational Politics

Political behavior is a common phenomenon within organizations, arising from interactions among members as they compete over and struggle for limited resources (Ferris & Kacmar, 1992). However, it is important to distinguish between political behavior—actions taken to gain power or influence—and perceived organizational politics, which refers to employees’ subjective perception that these behaviors shape decision-making and resource allocation (Ferris & Kacmar, 1992). Perceived organizational politics is not an objective measure of political actions but rather an individual’s cognitive interpretation of their work environment (Ferris et al., 2002).

Research identifies three primary dimensions of perceived organizational politics: (1) general politics, referring to perceptions that favoritism and informal alliances significantly influence organizational decision-making; (2) pay-and-promotion politics, which involve beliefs that compensation and career advancement decisions are politically motivated rather than merit-based; and (3) go-along-to-get-ahead politics, capturing the perception that conformity to certain social or political norms is necessary for career advancement (Ferris & Kacmar, 1992; Kacmar et al., 2011; Miller et al., 2008). These dimensions collectively reflect how employees interpret political dynamics within their organizations, influencing their perceptions of fairness, trust, and overall satisfaction (Ferris et al., 2002).

Among these dimensions, general politics is particularly influential in shaping job dissatisfaction and turnover intentions within bureaucratic contexts (Miller et al., 2008). In such settings, perceptions that informal alliances and favoritism influence decisions significantly undermine employee morale and satisfaction, ultimately increasing intentions to leave. Given their pronounced impact, we specifically address general political perceptions, aligning with previous research emphasizing their central role in public administration contexts. By focusing on general politics, we clarify the mechanisms through which political perceptions affect job satisfaction and turnover intentions. This targeted approach enhances understanding of how subjective perceptions, rather than specific pay-and-promotion or social conformity concerns, predominantly shape employee attitudes and organizational outcomes. Thus, we contribute clarity to existing literature by isolating and examining the most impactful dimension of perceived organizational politics in bureaucratic environments.

While scholars have widely studied public service motivation as a key driver of public employee retention, its effects are not uniform across different organizational settings. Public-sector environments vary significantly between Western and Asian administrative systems, particularly in terms of bureaucratic hierarchy, political influence in decision-making, and career advancement structures (Peters, 2018; Raadschelders, 2009).

In Western bureaucratic systems, particularly in the United States and Western Europe, public service institutions prioritize performance-based management, professional autonomy, and horizontal accountability (Welch & Wong, 1998). Employees in these settings expect transparent promotion processes, merit-based evaluations, and fairness in decision-making, and deviations from these norms—such as political favoritism—can erode organizational trust and increase turnover intentions (Vigoda-Gadot & Talmud, 2010). For instance, in the United Kingdom’s civil service, a shift toward performance-based promotions has been found to improve retention, whereas increased political influence over appointments has led to higher turnover rates (Andrews et al., 2017). In contrast, Asian public-sector organizations, including those in South Korea, China, and Japan, operate within seniority-based hierarchical systems, where long-term career stability, loyalty, and internal relationships play a greater role than short-term performance does (Choi, 2020; Debroux, 2022). In these environments, perceptions of fairness and political interference in personnel decisions may have an even greater impact on turnover intentions than in Western settings. This is particularly relevant in Confucian-influenced bureaucracies, where hierarchy, loyalty, and informal networks often shape career trajectories (Frederickson, 2002).

For example, in the South Korean civil service, employees who strongly identify with public values may initially tolerate bureaucratic inefficiencies. However, over time, exposure to political favoritism in promotions, opaque performance evaluations, or decision-making processes dominated by informal alliances can undermine their motivation and increase turnover intentions (Shim et al., 2017). Similarly, in China’s public administration, studies indicate that organizational loyalty and hierarchical stability can mitigate turnover, but when employees perceive political favoritism, turnover rates rise sharply (Ko & Han, 2013). Comparatively, in European civil services, high performance pressures, job market flexibility, and efficiency demands, rather than hierarchical constraints or internal political favoritism, often drive turnover intention (Van De Walle et al., 2015). This suggests that while public service motivation universally strengthens job commitment, its effectiveness in retaining employees depends heavily on perceptions of fairness, organizational transparency, and the level of political influence in career advancement.

While public service motivation has been widely recognized as a key factor in employee retention, its effectiveness in reducing turnover intention is not absolute and can vary depending on organizational conditions (Campbell & Im, 2016). One of the most critical contextual factors influencing this relationship is perceived organizational politics. Research has shown that employees’ perceptions of fairness, transparency, and political maneuvering may influence how they interpret their workplace experiences, influencing the extent to which intrinsic motivation, such as public service motivation, translates into long-term commitment (Park & Lee, 2020; Vigoda-Gadot & Kapun, 2005). In highly politicized environments, where promotions, resource distribution, and performance evaluations are perceived as being influenced more by favoritism than by meritocracy, the motivational effects of public service motivation may weaken, leading to higher turnover intentions among public employees (Wang et al., 2020).

Social exchange theory provides a useful framework for understanding this moderating effect (Cropanzano & Mitchell, 2005). It posits that employees engage in reciprocal relationships with their organizations based on trust and fairness—when they believe that their contributions are recognized and rewarded fairly, they reciprocate with greater organizational commitment and lower turnover intentions (Blau, 1964; Wayne et al., 1997). However, when employees perceive high levels of organizational politics, this trust-based exchange weakens, and they begin to question whether their organization reciprocates their commitment to public service. This can be particularly problematic for employees with high public service motivation because they may initially tolerate bureaucratic inefficiencies or lower salaries due to their intrinsic motivation, but over time, exposure to political favoritism in promotions and decision-making can erode their motivation and commitment.

For instance, in low-perceived organizational politics environments, where decision-making and promotions are perceived as fair and merit-based, employees with high public service motivation tend to remain committed because they believe that their contributions align with organizational values and are reciprocated with career progression and recognition (Tu et al., 2024). Employees with strong public service motivation are intrinsically driven to contribute to public service, and as long as they perceive fairness in the exchange relationship, they sustain their motivation and long-term commitment (Quratulain et al., 2019). However, in high-perceived organizational politics environments, where employees perceive that promotions, resource allocation, and rewards are based on favoritism rather than merit, the expected reciprocity in the social exchange relationship is broken (Wang et al., 2020). Even employees with high public service motivation may initially accept bureaucratic inefficiencies or lower salaries. However, repeated exposure to unfair practices—such as politically motivated promotions or resource hoarding by influential groups—can disillusion them over time (Vigoda-Gadot & Kapun, 2005). Over time, this perception of broken reciprocity could foster frustration and cynicism, leading employees to disengage from the organization despite their initial commitment to public service.

Although scholars have extensively researched public service motivation and its effects on turnover intention, the moderating role of perceived organizational politics in this relationship remains largely unexplored. While scholars have examined the independent effects of public service motivation and perceived organizational politics on job satisfaction and retention, few have investigated how perceived organizational politics might influence the connection between public service motivation and turnover intention in public sector environments. Empirical studies have shown that high levels of perceived organizational politics often exacerbate negative work attitudes and increase turnover intentions. This occurs because employees’ trust in their organization’s fairness and merit-based decision-making is undermined (De Clercq & Pereira, 2022; Harris et al., 2007). Research also indicates that public service motivation strongly predicts organizational commitment and job satisfaction, which ultimately leads to lower turnover intentions (Campbell & Im, 2016). However, in environments where political maneuvering and favoritism dominate, employees with high public service motivation may begin to feel that their efforts and contributions are not recognized or valued. This perception weakens the positive impact of public service motivation on turnover intention (Ha & Moon, 2023).

By incorporating these contextual insights, we emphasize the importance of organizational fairness and transparency in sustaining the benefits of public service motivation. Public servants engage in a reciprocal exchange with their organization. Their motivation and commitment are reinforced when their efforts are fairly recognized and rewarded (Blau, 1964; Cropanzano & Mitchell, 2005). However, in highly political environments where favoritism rather than merit drives career advancement, this reciprocity weakens. As a result, even highly motivated employees may reevaluate their commitment, leading to increased turnover intentions. Thus, perceived organizational politics serves as a key boundary condition, shaping whether public service motivation enhances retention or loses its effectiveness. Understanding this moderating effect is essential for creating a trust-based, transparent public-sector environment that supports employee retention. Based on this discussion, we propose the following hypotheses:

**Hypothesis 2:**  *Perceived organizational politics has a positive relationship with turnover intention.*

**Hypothesis 3:**  *Perceived organizational politics will moderate the negative relationship between public service motivation and turnover intention, such that this relationship is weaker when perceived organizational politics are higher compared to when they are lower.*

## 3. Model Specification

### 3.1. Research Design

**Figure 1:**
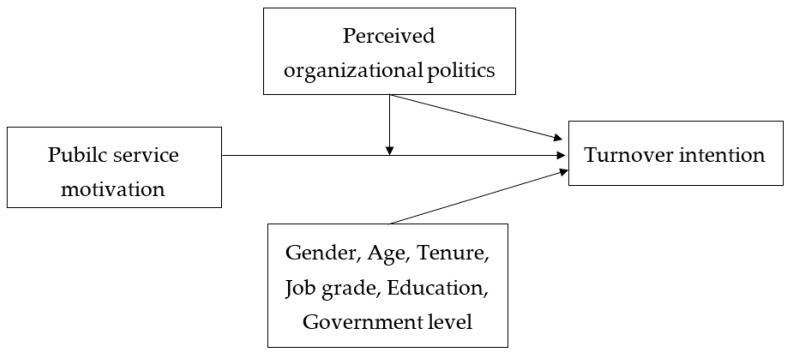
Hypothesized model.

Based on the theoretical discussions outlined above, we developed the research model presented in Figure 1. Our primary objective was to analyze how public service motivation affects turnover intention among public officials and to examine the moderating role of perceived organizational politics in this relationship. Public service motivation serves as the independent variable, turnover intention as the dependent variable, and perceived organizational politics as a moderating variable. In particular, we investigated the possibility that perceived organizational politics function as a critical contextual factor that weakens the negative relationship between public service motivation and turnover intention. Furthermore, demographic and organizational variables—including gender, age, length of service, position, education background, and employing agency—are included as control variables. We selected these variables based on prior research indicating their potential influence on turnover intention and public service motivation. Controlling these factors enhances analytic precision by accounting for alternative explanations. By systematically assessing public service motivation’s impact on turnover intention and conducting an in-depth analysis of perceived organizational politics’ moderating effect, we aim to offer concrete insights for managing public organizations.

### 3.2. Sample and Data Collection

To validate this study’s research model, data from the 2023 Korean Public Employee Viewpoints Survey, conducted by the Korea Institute of Public Administration (KIPA), were employed. KIPA is a Korean government research institute established to advance administration and policy, enhancing government operations’ efficiency and effectiveness through research on public policy and administrative systems. This survey aimed to examine the status of human resource management for public officials, investigate their subjective perceptions, and build an in-depth dataset.

The survey, conducted from 21 August to 30 September 2023, targeted 6444 general civil servants working in central government agencies, as well as in metropolitan and basic local government offices. The sampling method used a stratified cluster sampling approach, selecting participants from departments/teams within each institution. A minimum sample size was initially assigned to each institution, and the remaining samples were distributed using the power allocation method, a technique that allocates samples proportionally based on both the size of the stratum and its variance (or variability in responses). This prevents excessive concentration of samples in specific strata, ensuring a more representative sample across all groups.

To address potential errors resulting from the sampling process, a final weight was applied by multiplying the design weight by the nonresponse adjustment weight and post-stratification adjustment weight. Topics covered in the survey included work environment, personnel systems, organizational management, attitudes, and behaviors, with data collected via an email-based web survey.

The response rate of 6444 respondents, representing 100%, is based on the weighted analysis of the survey data rather than a simple count of respondents. The number 6444 is the adjusted total sample size, derived through statistical methods that account for the stratified sampling process and weighting procedure. After applying the power allocation method, the survey data were further refined by multiplying the design weight by the nonresponse adjustment weight and post-stratification adjustment weight. This adjustment ensures the sample accurately represents the entire population of public servants across different government sectors, addressing potential sampling and nonresponse biases. Thus, the weighted response rate of 100% means the number 6444 respondents accurately reflects the adjusted sample size, representing the broader population of public employees.

Table 1 summarizes the demographic characteristics of the survey respondents used in this study.

### 3.3. Measures

#### 3.3.1. Dependent Variable: Turnover Intention

In this study, the dependent variable was turnover intention, defined as an employee’s psychological state of voluntarily wanting to leave the organization under certain circumstances (Jung, 2010; Sun & Wang, 2017). We measured turnover intention using a single-item question: “I intend to leave my job if given the opportunity”. We assessed responses using a five-point Likert scale ranging from 1 (“Strongly disagree”) to 5 (“Strongly agree”). While multi-item scales are often preferred for their comprehensive assessment, single-item measures have been shown to be reliable when carefully designed and contextually appropriate (Chordiya, 2022; Cohen et al., 2016). We selected this single-item approach for its simplicity and alignment with previous research that has demonstrated its validity in capturing turnover intention (Mobley, 1977).

#### 3.3.2. Independent Variable: Public Service Motivation

Public service motivation is defined as an individual’s tendency to pursue the public interest and public values by serving the state and its citizens, rather than prioritizing personal benefits. It reflects a commitment to the public good and a willingness to help others (Perry & Wise, 1990). Public service motivation is commonly conceptualized as comprising four subdimensions: attraction to public policy, commitment to the public interest, compassion, and self-sacrifice (Perry, 1996). However, given that we aim to examine public service motivation as a holistic construct and its overall impact on turnover intention, we adopted a global measure of public service motivation that captures three of the four dimensions: commitment to the public interest, self-sacrifice, and compassion. This approach aligns with Wright et al. (2013), who found that a shortened public service motivation scale provides similar results on key correlates such as job satisfaction, job choice, and mission valence.

To measure public service motivation, we used the following five items: (1) “Serving my country and its people is extremely important to me”. (2) “Even if it makes me a subject of ridicule, I am willing to stand up for the rights of others”. (3) “For me, bringing about positive change in society holds greater significance than personal achievement”. (4) “I am ready to make considerable personal sacrifices if it serves the greater good of society”. (5) “In my daily life, I constantly reflect on how interdependent we all truly are”. These items align with previous research that conceptualizes public service motivation as a multidimensional yet cohesive construct (Kim & Vandenabeele, 2010). Scholars have validated each of these items in prior studies as appropriate indicators of the respective dimensions of public service motivation, ensuring a reliable and relevant measure for this study. We did not explicitly include the attraction to the public policy dimension in the measurement instrument because scholars in prior studies have suggested that overall public service motivation levels can still be reliably assessed using a composite index of the other three dimensions.

#### 3.3.3. Moderating Variable: Perceived Organizational Politics

Perceived organizational politics refers to the extent to which individuals perceive that self-serving behaviors rather than objective, merit-based criteria influence organizational decisions, resource allocation, and career advancement (Ferris & Kacmar, 1992; Hochwarter et al., 2020). These perceptions capture the subjective belief that political maneuvering, favoritism, or power imbalances play a role in shaping workplace dynamics rather than decisions being made in the organization’s overall best interest (Ferris & Kacmar, 1992). In this study, we conceptualize perceived organizational politics within the “general politics” dimension—one of the three established dimensions of perceived organizational politics, alongside pay-and-promotion politics and go-along-to-get-ahead politics (Ferris & Kacmar, 1992). General politics captures broad perceptions that favoritism, informal alliances, and self-serving behavior, rather than formal policies or meritocratic principles, influence organizational decision-making.

Accordingly, we treat perceived organizational politics in this study as a single composite index, given that both items capture different but interrelated aspects of general political perceptions. Specifically, we used two items to measure perceptions of organizational politics: (1) “There are people in our organization who present distorted information for their own gain” and (2) “There is a group in our organization that exercises considerable influence”. These items have been previously validated and reflect concerns about fairness, transparency, favoritism, and informal power structures within the organization. The first item evaluates the extent to which employees perceive manipulation of information for self-serving purposes, while the second item measures perceptions of concentrated power and informal influence within the organization. Both items are considered appropriate and valid indicators of general organizational politics, aligning with previous studies measuring perceived organizational politics (Ferris et al., 2000; Kacmar et al., 2011).

#### 3.3.4. Control Variables

In this study, we included demographic characteristics known to influence turnover intention as control variables to ensure a robust analysis. We controlled for gender (male = 0, female = 1) because scholars have suggested that women in public service may experience different career advancement opportunities and work–life balance concerns, influencing their turnover intentions (Campbell & Im, 2016). We included age (categorized as 20–29, 30–39, 40–49, and 50 and above), given evidence that younger employees exhibit higher turnover intentions due to greater career mobility, whereas older employees tend to remain in stable public-sector roles (Cho & Lewis, 2012). We considered tenure (1 = fewer than five years, 2 = 6–10 years, 3 = 11–15 years, 4 = 16–20 years, 5 = 21–25 years, and 6 = 26 years or more) because employees with longer lengths of service are more likely to leave, while those with shorter tenure exhibit stronger organizational commitment (Suzuki & Hur, 2020). We controlled for job rank (Grades 1–4, Grade 5, Grades 6–7, and Grades 8–9) because research indicates that employees in higher-ranking positions enjoy greater job security and autonomy, leading to lower turnover rates (Ha & Moon, 2023). We included education level (ranging from high school or below to doctorate) because individuals with higher education tend to have greater job mobility and external opportunities, increasing their likelihood of turnover (Moynihan & Landuyt, 2008). Lastly, we controlled for organizational affiliation (central government = 1, local government = 0) because studies indicate that central and local government employees may have different turnover patterns due to variations in job stability and career development opportunities (Campbell et al., 2014). By incorporating these control variables, we account for individual and structural factors that have been empirically linked to turnover intention in public-sector settings, ensuring a more precise assessment of the moderating role of perceived organizational politics in the relationship between public service motivation and turnover intention.

#### 3.3.5. Measurement Reliability and Validity

To verify the selected survey items’ validity, we conducted an exploratory factor analysis using orthogonal factor rotation. We extracted factors based on an eigenvalue of 1.0 or higher and retained factor loadings at 0.5 or above (see Table 2). Excluding the single item measuring turnover intention, we grouped the remaining seven survey items into two factors, which aligned with the public service motivation and perceived organizational politics concepts presented in this study, thereby confirming the validity of each variable’s survey item composition. We assessed reliability using Cronbach’s α, a commonly employed method to evaluate internal consistency for multi-item scales. A Cronbach’s α value of 0.7 or higher is generally viewed as indicative of high internal consistency. The analysis revealed that public service motivation, measured with five items, had a Cronbach’s α of 0.881, confirming its reliability. For perceived organizational politics, measured with two items, we assessed inter-item correlation instead of Cronbach’s α. Cronbach’s α is not appropriate for two-item scales because it can produce biased or unstable estimates (Eisinga et al., 2013). Instead, inter-item correlation is recommended for two-item scales (Rammstedt & Beierlein, 2014). The inter-item correlation for the two items of perceived organizational politics was 0.630.

### 3.4. Methodology

While some scholars treat ordinal outcomes as continuous variables and apply linear regression (Ananth & Kleinbaum, 1997; McCullagh, 1980), this approach assumes that the distances between response categories are equal, which may not always hold in subjective measures such as turnover intention. To ensure that the model appropriately captures the ordinal nature of the dependent variable, we initially employed an ordinal logistic regression approach (Long & Freese, 2006). However, a likelihood ratio test rejected the parallel regression assumption (χ² = 151.50, *p* < 0.01), indicating that a standard ordinal model was unsuitable. To address this, we adopted stereotype logistic regression as a more flexible alternative. Unlike ordinal logistic regression, which assumes proportional odds across categories, stereotype logistic regression allows for different slopes for each category of the dependent variable while maintaining the ordinal structure. This method is particularly suitable when the proportional odds assumption is violated and allows for varying category distances, mitigating the potential distortions that could arise if turnover intention were treated as a strictly interval-scaled variable.

## 4. Results

Table 3 presents the descriptive statistics and correlation coefficients for this study’s major variables. The correlation analysis revealed that public service motivation has a more negative correlation with turnover intention (r = −0.204, *p* < 0.01) than other demographic variables such as age (r = −0.210, *p* < 0.01) and length of service (r = −0.218, *p* < 0.01). This suggests that while tenure and age influence retention, the effect of public service motivation is comparable in magnitude to these demographic factors. In contrast, education level exhibits a positive correlation with turnover intention (r = 0.145, *p* < 0.01), indicating that higher-educated employees are more likely to consider leaving, possibly due to greater career mobility. Given these results, public service motivation appears to be as influential as key demographic factors in shaping turnover intention, reinforcing its role as a strong predictor of public employee retention.

To establish a baseline model, we designed Model 1 first to assess the effects of demographic and organizational factors on turnover intention as well as the direct effect of perceived organizational politics before introducing the study’s primary independent variable, public service motivation, in Model 2. This approach ensures that other factors do not confound any observed relationship between public service motivation and turnover intention in later models, providing a clearer estimation of public service motivation’s unique contribution. Model 3 then examines the interaction effect between public service motivation and perceived organizational politics, testing how the level of perceived organizational politics influences the relationship between public service motivation and turnover intention.

The results for Model 1 in Table 4 show that age (β = −0.514, *p* < 0.01) and length of service (β = −0.311, *p* < 0.01) both relate negatively to turnover intention, suggesting that those with more experience within the organization are more inclined to stay. Factors such as long-term career development, pension benefits, and established networks may function as turnover deterrents. However, job rank (β = 0.276, *p* < 0.05) and education level (β = 0.606, *p* < 0.01) were positively associated with turnover intention, implying that officials in higher positions or with higher education levels are more likely to consider leaving in search of better opportunities, possibly because higher-level officials experience greater political pressures within the organization or highly educated officials more actively examine external career transitions. Furthermore, we found organizational affiliation (β = −0.348, *p* < 0.01) to have a negative relationship with turnover intention, indicating that officials within central government agencies tend to have lower turnover intentions than those in local governments, possibly due to the relatively stable working environments and better career development opportunities in central agencies. Finally, perceived organizational politics (β = 0.703, *p* < 0.01) exhibited a significant positive relationship with turnover intention, suggesting that individuals who strongly perceive political factors—such as unfair decision-making, biased resource allocation, and power used for personal gain—tend to experience decreased trust in the organization and reduced commitment to long-term service. This finding supports Hypothesis 2, which posited that higher levels of perceived organizational politics would be positively associated with turnover intention. By demonstrating this relationship, we confirm that organizational politics can contribute to employees’ intentions to leave, highlighting the negative impact of perceived political behaviors on organizational commitment.

The results for Model 2 indicate that the independent variable, public service motivation, exerts a significant negative effect on turnover intention (β = −0.995, *p* < 0.01), supporting Hypothesis 1, which states that higher public service motivation leads to lower turnover intention.

**Figure 2:**
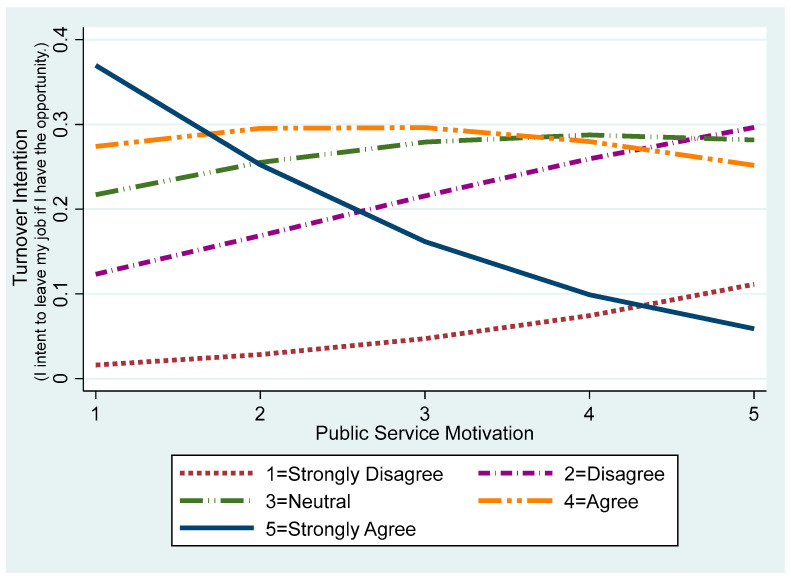
Predicted probability of turnover intention by level of public service motivation.

Figure 2 visualizes the impact of public service motivation on turnover intention by illustrating predicted probabilities across each response category. First, at higher turnover intention levels (4 = agree, 5 = strongly agree), the likelihood of these responses decreases as public service motivation increases. Notably, the probability of “strongly agree (5)” drops sharply with increasing public service motivation, indicating that individuals with high levels of public service motivation are markedly less likely to consider leaving. Conversely, lower turnover intention levels (1 = strongly disagree, 2 = disagree) gradually increase as public service motivation grows, suggesting that stronger public service motivation fosters greater attachment and commitment to the organization. Meanwhile, responses of “neutral (3)” show little change as public service motivation increases, suggesting that public service motivation’s effect might be limited at certain levels. For “agree (4)”, the probability also decreases, but more gradually than for “strongly agree (5)”, suggesting that public service motivation’s turnover-reducing effect may vary across different levels of intent to leave.

While public service motivation significantly reduces turnover intention, its effect is comparable to that of key demographic variables such as age and length of service. Employees with longer tenure tend to stay, likely due to career stability and pension benefits, while younger or highly educated employees show greater turnover intentions, possibly seeking career advancement outside the public sector. However, public service motivation’s effect remains significant even after accounting for these factors, suggesting that public service motivation functions as an intrinsic commitment mechanism that influences employees beyond structural career incentives. This underscores the importance of fostering a work environment that aligns with employees’ public service values to sustain long-term commitment.

**Figure 3:**
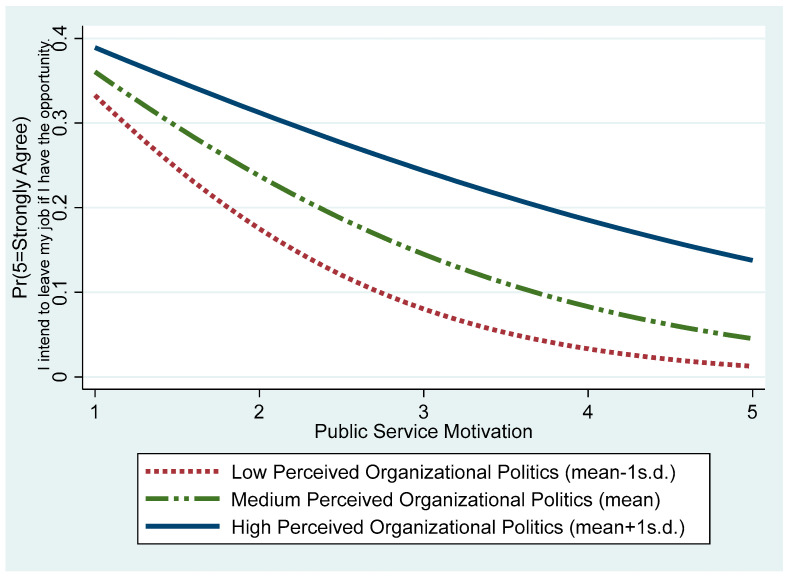
Probability of “strongly agree” on turnover intention: Varying public service motivation and perceived organizational politics levels.

**Figure 4:**
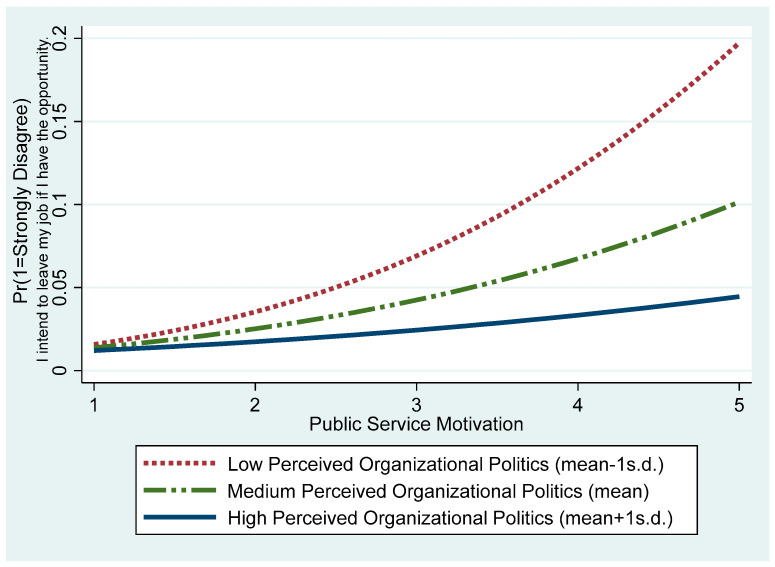
Probability of “strongly disagree” on turnover intention: Varying public service motivation and perceived organizational politics levels.

The Model 3 results support Hypothesis 3, indicating that perceived organizational politics moderates the relationship between public service motivation and turnover intention (β = 0.247, *p* < 0.01). In other words, as awareness of political dynamics within the organization increases, the negative effect of public service motivation on turnover intention weakens. Specifically, Figure 3 visually depicts public service motivation’s marginal effect on turnover intention under varying perceived organizational politics levels. When perceived organizational politics are low (mean − 1 SD), turnover intention in the category “strongly agree” declines sharply as public service motivation increases. However, when perceived organizational politics are high (mean + 1 SD), the rate of decline becomes more gradual. Figure 4 illustrates how public service motivation influences the turnover intention of employees who responded “strongly disagree” under varying levels of perceived organizational politics. In contexts with low perceived organizational politics, the negative effect of public service motivation is relatively modest, but in contexts with high perceived organizational politics, this effect weakens, resulting in a higher probability of turnover intention.

Interpreting these findings through social exchange theory suggests that the relationship between an organization and its members is based on reciprocity and trust. When an organization maintains fairness, employees reciprocate with increased commitment and dedication. However, as perceived organizational politics increases, employees may perceive that the organization does not provide a conducive environment for upholding their public values, and they become less confident about receiving fair evaluations and rewards. This weakens the social exchange relationship with the organization and creates an environment where public service motivation struggles to function as an intrinsic motivator, thereby raising the likelihood of turnover. In other words, even public officials with high public service motivation may lose trust in the organization if political elements are pervasive, ultimately rendering them more inclined to consider leaving.

## 5. Discussion

### 5.1. Theoretical Implications

In this study, we set out to examine how public service motivation affects turnover intention in public organizations and to verify perceived organizational politics as a moderating factor in this relationship. Drawing on social exchange theory, we highlight the importance of fairness, trust, and reciprocity in the employee-organization relationship (Cropanzano & Mitchell, 2005). The analysis confirmed that higher levels of public service motivation are associated with lower turnover intention, supporting the first hypothesis. We also found perceived organizational politics to be positively related to turnover intention, suggesting that as employees perceive higher levels of organizational politics, their intention to leave the organization increases. Moreover, the study revealed that perceived organizational politics weakens the negative relationship between public service motivation and turnover intention, supporting the third hypothesis. This suggests that the presence of high organizational politics diminishes the positive effect of public service motivation on reducing turnover intention.

Building on social exchange theory, this study emphasizes that employees’ turnover intentions are influenced not only by their intrinsic motivation but also by their perceptions of fairness and trust in the organization. When fairness is compromised due to political dynamics, employees’ sense of reciprocity weakens, which can undermine their organizational commitment even when they have high public service motivation (Quratulain et al., 2019). This perspective extends social exchange theory by demonstrating that contextual factors such as organizational politics can alter the expected benefits of public service motivation in reducing turnover intention (Wang et al., 2020). Consequently, the moderating role of perceived organizational politics introduces a crucial dimension to social exchange theory, highlighting the need to consider organizational politics when evaluating public service motivation’s effectiveness as a retention mechanism.

Previous studies across various contexts have consistently shown that public service motivation positively influences public-sector employees’ attitudes and behaviors, such as job satisfaction and organizational commitment (Campbell & Im, 2016; Homberg et al., 2015; Vandenabeele, 2007). Comparative research between public and private organizations has demonstrated that public service motivation strengthens organizational commitment and reduces turnover intention. However, prior scholars have not fully accounted for the influence of organizational politics on this relationship. By empirically testing how perceived organizational politics interacts with public service motivation to shape turnover intention, we address a contextual factor that past scholars have largely neglected. These findings underscore that public service motivation is not merely an individual disposition but a motivation that the organizational environment significantly shapes.

This study also contributes to the literature by focusing on the direct effect of public service motivation on employee retention. Individuals with high public service motivation tend to stay longer in organizations, driven by their sense of duty and commitment to public service rather than short-term rewards. While previous scholars have primarily examined how public service motivation enhances job satisfaction, organizational commitment, and public service performance, this study shifts the focus to its retention effect. This distinction highlights how public service motivation functions not only as an individual motivational factor but also as a force that interacts with broader organizational dynamics, such as fairness and trust, to influence employees’ likelihood of staying.

Finally, this study expands the scope of public service motivation research by integrating the moderating role of perceived organizational politics while emphasizing the validity of social exchange theory. While prior scholars have largely overlooked the role of organizational context, we provide empirical evidence on both the direct effect and the moderating effect of perceived organizational politics through the lens of social exchange theory. We found that perceived organizational politics has a positive direct effect on turnover intention, suggesting that as employees perceive higher levels of organizational politics, their intention to leave the organization increases. This is consistent with social exchange theory, which highlights the importance of fairness and reciprocity in the employee-organization relationship. When employees perceive organizational politics as high, they may feel that social exchange is unfair or not mutually beneficial, leading to an increased desire to leave.

### 5.2. Practical Implications

To enhance employee retention in public organizations, it is essential to adopt strategies that mitigate perceived organizational politics while reinforcing public service motivation. While intrinsic motivation plays a critical role in employee commitment, it is not sufficient on its own. Organizations must actively foster a work environment characterized by fairness, transparency, and trust to ensure that public service motivation translates into long-term retention. This requires a comprehensive approach spanning recruitment, job design, leadership development, and performance management.

One of the most fundamental steps in this process is ensuring that public-sector organizations attract and retain individuals with strong public service motivation from the outset. Effective recruitment and selection processes are crucial for identifying candidates who are not only qualified but also deeply committed to public service values. The practical implications of this study focus on refining hiring and retention strategies to strengthen public service motivation within organizations. To recruit talent with high public service motivation, organizations must go beyond hiring practices based solely on academic credentials or job experience and introduce multifaceted procedures that assess commitment to public values and ethical responsibility. First, during the document-screening stage, they must more thoroughly assess applicants’ efforts to realize public values. Rather than simply verifying volunteer experience, organizations should conduct both quantitative and qualitative evaluations of nonprofit activities, public policy participation, and community problem-solving efforts. For example, in the selection process, organizations could examine whether applicants have driven actual change through specific policy proposals or by playing a leading role in civic society work. Second, in the interview process, organizations should employ more sophisticated situational judgment tests to thoroughly evaluate the core components of public service motivation, namely the desire to contribute to the public good, self-sacrifice, and ethical responsibility. By examining how applicants would resolve real ethical dilemmas they might face within a public organization, it is possible to gauge their genuine values and attitudes rather than receive only textbook answers. A question such as “How would you respond if a particular interest group exerted pressure during policy implementation?” can help assess an applicant’s commitment to prioritizing public interests. Third, strengthening person-organization fit evaluation is crucial for measuring how closely applicants resonate with the organization’s public values and vision. Tools such as public service motivation-based personality tests or values-based interviews may be adopted, and organizations can also offer training and mentoring on public sector values to help new hires internalize them more effectively. By systematically introducing these selection methods, public-sector organizations can secure individuals likely to remain for the long term and ensure that public service motivation is consistently strengthened within the organization.

Maintaining and reinforcing public service motivation requires well-structured motivational strategies at the organizational level. First, a job redesign emphasizing fulfilling public values should clarify to officials how their responsibilities contribute to the public good. Rather than viewing their tasks as merely administrative, employees should be able to witness the positive impact their policy work has on citizens. When civil servants recognize that they directly contribute to citizens’ welfare and public service quality, their public service motivation strengthens naturally. Second, leadership that promotes public service motivation must be developed. By combining ethical and transformational leadership, the organization’s managers should consistently practice public values themselves and embed them into the culture. For example, if leaders give directives and expand citizen participation in policymaking or implement fair evaluation systems, employees will develop a stronger sense of mission toward public service. Third, organizations should operate programs that broaden employees’ opportunities to contribute socially. If officials regularly engage in community service or policy research projects, they experience how their work can affect social change more concretely. Fourth, public service motivation must be incorporated into the performance evaluation and reward system. Instead of relying solely on outcome-based assessments, organizations should incorporate criteria that measure efforts to improve public service quality, such as citizen satisfaction and social value creation. This encourages employees to maintain public service motivation-oriented attitudes over time, ultimately enhancing their sense of job accomplishment and organizational commitment.

Reducing perceived organizational politics requires strategies that enhance fairness and transparency within the organization, minimizing opportunities for political maneuvering. First, decision-making transparency must be improved. Before making key decisions such as promotions, job rotations, or performance evaluations, organizations should disclose objective criteria and provide employees with a channel for filing objections to outcomes. Introducing an AI-based evaluation system can also curb political interference in personnel processes. Second, fair resource allocation systems must be established. Organizational resources—such as budgets, project opportunities, and promotions—should not be allocated toward particular groups, and a regular audit system should verify that distribution principles are upheld. For example, if promotion opportunities repeatedly focus on a specific division or individual, an external committee of experts can be formed to conduct impartial reviews, thereby reducing political interference. Third, leadership that curbs political behavior should be strengthened. Instead of favoring select groups, higher-level managers should strive to embed fairness and public values into the organizational culture. This can be achieved by enhancing leadership training and incorporating fairness and ethical responsibility into leaders’ performance evaluations. Fourth, whistleblower systems and protective mechanisms should be strengthened to prevent unjust political acts, such as nepotism and connections-based promotions. By establishing an anonymous reporting channel and enacting legal and institutional safeguards for whistleblowers, organizations can deter unethical political conduct, thereby strengthening organizational trust and fairness.

### 5.3. International Context and Applicability

While this study primarily focuses on the Korean public sector, it is important to recognize the differences in public sector recruitment and retention processes across countries. The Korean civil service system is highly structured, with recruitment primarily based on competitive civil service examinations, a hierarchical promotion system emphasizing seniority, and considerable political influence in personnel decisions. This structure fosters long-term career commitment but may also create rigid organizational dynamics that amplify how organizational politics affect turnover intention. In contrast, countries with performance-based promotion systems, such as the United States and the United Kingdom, often emphasize individual competencies, annual evaluations, and managerial discretion in promotions (Hodder, 2015; Horton, 2011). In such settings, public service motivation may interact differently with career advancement opportunities, potentially reducing turnover intention if employees perceive a clear link between performance and rewards. Similarly, in Nordic countries, where collaborative governance and lower political interference are key features of public administration, perceived organizational politics may have a weaker impact on turnover intention because decision-making processes are more transparent and consensus-driven.

Public sector recruitment in Korea also involves a strict, examination-based entry system, where candidates prepare for years before securing a civil service position. This high entry barrier fosters strong occupational commitment but may also lead to frustration if promotion opportunities are perceived as politically influenced rather than meritocratic. In contrast, continental European countries such as Germany and France implement hybrid public sector recruitment systems that combine competitive examinations with structured career progression, albeit with differing degrees of centralization (Bekke & Meer, 2000). Germany follows a decentralized model where individual ministries autonomously recruit and manage personnel, while France maintains a more centralized system with competitive entry through institutions such as the National School of Administration. These institutional differences shape career motivations and perceptions of political dynamics within public administration.

Therefore, while the findings of this study provide meaningful insights into Korean public organizations, further research is necessary to examine how variations in recruitment mechanisms, promotion criteria, and political influence affect the relationship between public service motivation, organizational politics, and turnover intention in diverse administrative contexts. A comparative analysis across different governance models would offer a more nuanced understanding of how public service motivation and workplace politics influence public sector retention globally.

### 5.4. Limitations and Future Research

Although this study provides meaningful theoretical and policy suggestions for managing public organizations, several limitations exist. First, while we chose perceived organizational politics as the moderating variable, we did not capture a broader range of environmental factors within public organizations. For example, organizational fairness (procedural, distributive, and interactional), perceived organizational support, and leadership styles (ethical leadership, servant leadership, etc.) may also moderate the relationship between public service motivation and turnover intention, but we did not include them here. Future scholars should expand the analysis to incorporate these and other organizational variables to examine in greater detail the conditions that encourage or hinder the development of public service motivation.

Second, we did not address the multidimensional nature of public service motivation. Rather than being a unitary concept, public service motivation comprises rational, normative, and affective motivations. However, we treated it as a single dimension, meaning potential differences in how each motivation type affects turnover intention—particularly in interactions with perceived organizational politics—remain underexamined. In the future, researchers could measure these subcomponents more precisely and analyze their distinct effects on turnover intention under varying organizational political conditions. This would clarify the detailed mechanisms of influence of public service motivation and allow for more targeted motivational strategies within public organizations.

Third, while we adopted a global measure of public service motivation to assess its overall impact, future researchers should further explore the independent effects of the four subdimensions of public service motivation. Given that we did not directly incorporate attraction to public policy into the measurement instrument, researchers could examine whether this dimension plays a distinct role in shaping public employees’ attitudes and behaviors. A more fine-grained analysis of how each public service motivation subdimension differentially influences organizational commitment, job performance, and turnover intention would provide deeper theoretical and practical insights into the mechanisms underlying public service motivation.

Similarly, we measured turnover intention using a single-item measure. While this approach offers simplicity and has been validated in prior research, it may not fully capture the complexity of employees’ turnover intentions. Future studies could benefit from researchers’ employing multi-item measures to further validate the reliability of the turnover intention construct (Hom et al., 1984). A more comprehensive assessment using multiple indicators would allow for a more nuanced understanding of employees’ intent to leave and provide greater reliability in measuring turnover-related attitudes and behaviors.

While this study focuses on the general politics dimension of perceived organizational politics, future researchers should examine the differential effects of its three established subdimensions: general politics, pay-and-promotion politics, and go-along-to-get-ahead politics. Each of these dimensions reflects distinct yet interrelated aspects of political perceptions—such as broad favoritism (general politics), politically influenced career advancement (pay-and-promotion politics), and pressure to conform for career benefits (go-along-to-get-ahead politics). Understanding how these dimensions uniquely shape employees’ attitudes, workplace experiences, and turnover intentions would provide a more nuanced perspective on the role of organizational politics in public sector retention.

To achieve this, future researchers should also refine the measurement of perceived organizational politics by incorporating a more comprehensive set of items. While we relied on two items assessing general political perceptions, expanding the item set in alignment with validated perceived organizational politics scales (Ferris & Kacmar, 1992) would enhance construct validity and enable a more precise examination of how different facets of organizational politics influence employee behavior. A deeper analysis of these subdimensions, coupled with improved measurement instruments, would contribute to a more thorough understanding of how organizational politics manifests across different workplace contexts and inform targeted strategies for mitigating its negative effects.

Moreover, while in this study we applied social exchange theory as its primary theoretical framework to explain how perceived organizational politics moderates the relationship between public service motivation and turnover intention, it is important to acknowledge that alternative turnover theories may offer valuable perspectives. Established models such as the theory of organizational equilibrium (March & Simon, 1958), the theory of planned behavior (Ajzen, 1991), the unfolding model of turnover (Lee & Mitchell, 1994), and the job embeddedness model (Mitchell et al., 2001) provide additional conceptual lenses through which the mechanisms underlying employee turnover can be examined. These frameworks emphasize a variety of psychological and contextual factors influencing turnover decisions, including job satisfaction, behavioral intentions, and external labor market conditions. While social exchange theory effectively captures the role of fairness and reciprocity in shaping public service motivation and turnover dynamics, future studies may benefit from integrating these alternative theoretical models to develop a more comprehensive understanding of turnover processes in the public sector.

Finally, because we used cross-sectional survey data, we could not capture public service motivation or turnover intention’s progression over time. Individual public service motivation may evolve with career development and organizational experiences, and perceived organizational politics also may shift. However, using data collected at a single point in time means that we could not thoroughly examine the dynamic relationship between public service motivation and turnover intention. Future scholars should utilize longitudinal data to observe how public service motivation changes and influences turnover intention in the long run. Employing in-depth interviews with various stakeholders or experimental research designs could refine causal analysis further and yield more practical insights for public-sector human resource management and policy formulation.

## 6. Conclusions

This study provides valuable insights into the complex relationship between public service motivation and turnover intention in public organizations, offering a significant theoretical and practical contribution to the field. By incorporating perceived organizational politics as a moderating factor, it extends the application of social exchange theory and demonstrates that while public service motivation typically reduces turnover intention, its effects are significantly weakened in environments where organizational politics undermine fairness and trust. This is a crucial addition to existing research because it highlights that public service motivation’s impact on retention is not solely an individual characteristic but one that operates within the broader organizational context. By showing how high levels of perceived organizational politics can diminish the benefits of public service motivation, this study underscores the importance of creating work environments that are fair, transparent, and free from undue political influence to fully leverage the potential of public service motivation.

The practical implications of this research are far-reaching for public sector organizations aiming to recruit and retain motivated individuals. The study suggests that recruitment should go beyond traditional metrics, incorporating assessments of candidates’ commitment to public values, ethical responsibility, and organizational alignment. Furthermore, fostering a culture of fairness and transparency, coupled with ethical leadership, is essential for reinforcing the positive effects of public service motivation. This research also lays the groundwork for future studies by pointing out key limitations, such as the need to explore the multidimensional nature of public service motivation and the different subdimensions of perceived organizational politics, offering future research avenues that can deepen our understanding of how public service motivation and organizational politics influence retention in more nuanced ways. Ultimately, this study emphasizes the need for public organizations to adopt strategies that address both individual motivation and organizational dynamics, creating environments where employees are motivated to stay and contribute meaningfully to the public good.

## Figures and Tables

**Table 1:** Demographic information on the sample.

Category	Subcategory	Number of Cases	Percentage (%)
Gender	Male	3444	53.45
	Female	3000	46.55
Age	≤29	700	10.86
	30–39	2472	38.36
	40–49	1999	31.02
	≥50	1273	19.75
Tenure	≤5	1924	29.86
	6–10	1499	23.26
	11–15	808	12.54
	16–20	985	15.29
	21–25	393	6.10
	≥26	835	12.96
Job grade	1–4	200	3.10
	5	917	14.23
	6–7	3664	56.86
	8–9	1663	25.81
Education	High school or below	319	4.95
	Associate’s degree	315	4.89
	Bachelor’s degree	5053	78.41
	Master’s degree	646	10.02
	Doctorate	111	1.72
Government level	Central	1673	44.35
	Local	2099	55.65

**Table 2:** Factor loadings.

Latent Variables	Survey Items	Factor 1	Factor 2
Public service motivation	■ Serving my country and its people is extremely important to me	0.829	-
	■ Even if it makes me a subject of ridicule, I am willing to stand up for the rights of others	0.850	-
	■ For me, bringing about positive change in society holds greater significance than personal achievement	0.860	-
	■ I am ready to make considerable personal sacrifices if it serves the greater good of society	0.845	-
	■ In my daily life, I constantly reflect on how interdependent we all are	0.719	-
Perceived organizational politics	■ There are people in our organization who present distorted information for their own gain	-	0.905
	■ There is a group in our organization that exercises considerable influence	-	0.898

**Table 3:** Descriptive statistics and correlations.

	(1)	(2)	(3)	(4)	(5)	(6)	(7)	(8)	(9)
(1)	1								
(2)	−0.204 ***	1							
(3)	0.141 ***	−0.115 ***	1						
(4)	0.032 ***	−0.113 ***	0.077 ***	1					
(5)	−0.210 ***	0.252 ***	0.018 *	−0.143 ***	1				
(6)	−0.218 ***	0.237 ***	0.012	−0.051 ***	0.840 ***	1			
(7)	0.145 ***	−0.163 ***	−0.012 *	0.099 ***	−0.498 ***	−0.514 ***	1		
(8)	0.032 *	0.105 ***	−0.007	−0.007	0.223 ***	0.121 ***	−0.283 ***	1	
(9)	−0.025	0.029 *	−0.039 **	0.004	−0.042 **	−0.090 ***	−0.234 ***	0.152 ***	1
mean	3.331	3.139	2.906	0.466	2.597	2.834	3.054	2.987	0.556
s.d.	1.141	0.727	0.874	0.499	0.924	1.721	0.722	0.645	0.497

***Note***: * *p* < 0.1; ** *p* < 0.05; *** *p* < 0.01; (1) = Turnover intention; (2) = Public service motivation; (3) = Perceived organizational politics; (4) = Gender; (5) = Age; (6) = Tenure; (7) = Job grade; (8) = Education; (9) = Government level; s.d. = Standard deviation.

**Table 4:** Stereotype logistic models for hypothesized relationships.

	Model 1		Model 2		Model 3	
	β		β		β	
	(S.E.)		(S.E.)		(S.E.)	
Gender (female = 1)	−0.057		−0.153		−0.145	
	(0.135)		(0.140)		(0.139)	
Age	−0.514	***	−0.426	***	−0.413	***
	(0.137)		(0.143)		(0.141)	
Tenure	−0.311	***	−0.299	***	−0.305	***
	(0.076)		(0.079)		(0.078)	
Job grade	0.276	**	0.277	**	0.266	**
	(0.117)		(0.122)		(0.121)	
Education	0.606	***	0.703	***	0.703	***
	(0.110)		(0.116)		(0.116)	
Government level	−0.348	**	−0.282	*	−0.278	*
	(0.143)		(0.149)		(0.147)	
Perceived organizational politics (A)	0.703	***	0.662	***	−0.125	
	(0.085)		(0.085)		(0.280)	
Public service motivation (B)			−0.995	***	−1.735	***
			(0.106)		(0.280)	
(A) × (B)					0.247	***
					(0.085)	
$\phi_{1}$	1.000		1.000		1.000	
$\phi_{2}$	0.760		0.728		0.737	
$\phi_{3}$	0.542		0.556		0.578	
$\phi_{4}$	0.431		0.465		0.486	
$\theta_{1}$	1.094		−1.608		−3.973	
$\theta_{2}$	2.088		0.123		−1.620	
$\theta_{3}$	1.874		0.474		−0.898	
$\theta_{4}$	1.676		0.567		−0.587	
Log likelihood	−5498.421		−5445.518		−5541.384	
Wald $\chi^{2}$	199.320	***	275.210	***	291.280	***

***Note***: * *p* < 0.1; ** *p* < 0.05; *** *p* < 0.01; S.E. = robust standard error.

## Data Availability

The data used for this study are available at https://www.kipa.re.kr/site/kipa/stadb/selectBaseDBFList.do (accessed on 20 December 2024). Permission to use data must be obtained from KIPA.
